# Alternative Dietary Fiber Sources in Companion Animal Nutrition

**DOI:** 10.3390/nu5083099

**Published:** 2013-08-06

**Authors:** Maria R. C. de Godoy, Katherine R. Kerr, George C. Fahey

**Affiliations:** Department of Animal Sciences, University of Illinois, 1207 W, Gregory Drive, Urbana, IL 61801, USA; E-Mails: krkerr2@illinois.edu (K.R.K.); gcfahey@illinois.edu (G.C.F.J.)

**Keywords:** companion animal, beet pulp, cellulose, corn fiber, fruit fiber, rice bran, whole grains

## Abstract

The US has a pet population of approximately 70 million dogs and 74 million cats. Humans have developed a strong emotional bond with companion animals. As a consequence, pet owners seek ways to improve health, quality of life and longevity of their pets. Advances in canine and feline nutrition have contributed to improved longevity and well-being. Dietary fibers have gained renewed interest in the pet food industry, due to their important role in affecting laxation and stool quality. More recently, because of increased awareness of the beneficial effects of dietary fibers in health, as well as the popularity of functional foods and holistic and natural diets, alternative and novel carbohydrates have become widespread in human and pet nutrition. Fiber sources from cereal grains, whole grains and fruits have received increasing attention by the pet food industry and pet owners. While limited scientific information is available on the nutritional and nutraceutical properties of alternative fiber sources, studies indicate that corn fiber is an efficacious fiber source for pets, showing no detrimental effects on palatability or nutrient digestibility, while lowering the glycemic response in adult dogs. Fruit fiber and pomaces have good water-binding properties, which may be advantageous in wet pet food production, where a greater water content is required, along with low water activity and a firm texture of the final product. Rice bran is a palatable fiber source for dogs and may be an economical alternative to prebiotic supplementation of pet foods. However, it increases the dietary requirement of taurine in cats. Barley up to 40% in a dry extruded diet is well tolerated by adult dogs. In addition, consumption of complex carbohydrates has shown a protective effect on cardiovascular disease and oxidative stress. Alternative fiber sources are suitable ingredients for pet foods. They have been shown to be nutritionally adequate and to have potential nutraceutical properties.

## 1. Introduction

At year-end of 2011, the companion animal population in the US was comprised of approximately 70 million dogs and 74 million cats, with nearly 67% of households owning at least one pet [1]. Currently, the role played by dogs and cats in the American household is very different from when they were first domesticated. During this process, a strong emotional human-animal bond was developed, and pet animals have assumed a pivotal role in family systems and society. Pets became a source of emotional, therapeutic and psychological support, were brought inside the home and started sharing the same environment, food and lifestyle as their owners.

The increasing importance of companion animals is noted, as 63% of the American pet owners considered dogs and cats to be family members, and another 35% consider their pets as companions [1]. As a consequence, pet owners seek ways to improve health, quality of life and longevity of their companion animals. Advances in veterinary medicine have helped to increase the life span of dogs and cats. However, the growing knowledge in canine and feline nutrition also has contributed to improve longevity and well-being. Among the nutrient categories, dietary fibers have gained renewed interest in the pet food industry, as they play an important role in modulating bowel movement, influencing immune function and gut microbiota profile, diluting caloric density, contributing to weight loss and, indirectly, ameliorating the incidence of obesity and diabetes mellitus in the pet population.

Traditional sources of dietary fibers used in pet foods include beet pulp and cellulose. Beet pulp contains both insoluble and soluble fiber components in a desirable ratio. Cellulose is composed of insoluble and poorly fermentable fiber. In this review, the term insoluble and soluble fiber will be used, because the literature reviewed refers to these terms; however, the Dietary Reference Intakes (DRI) fiber report has replaced these terms with fermentable and viscosity [2]. In general, the beneficial effects of fermentable and soluble fibers on health are related to increased digesta viscosity, decreased gastric emptying, increased satiety, reduced rate of glucose uptake, lower blood cholesterol concentrations and promotion of gut commensal bacteria growth [3,4,5,6]. Conversely, non-fermentable fibers may decrease gastric transit time, dilute diet caloric density, increase fecal bulk and moisture and aid in laxation [7].

More recently, because of the increased awareness of the beneficial effects of dietary fibers in health, as well as the popularity of functional foods and holistic and natural diets, alternative and novel carbohydrates, here defined as fiber sources not typically used in diet matrixes, have become widespread in human and pet nutrition. Consequently, low digestible carbohydrates and cereal grains with a low-glycemic index and (or) rich in fermentable and soluble fibers, such as soluble corn and fruit fibers, and whole grains, like barley (*Hordeum vulgare*) and oats (*Avena sativa*), have received attention by the pet food industry. Aside from their nutritional and potential nutraceutical values, these ingredients have positive tag appeal for pet owners who anthropomorphize their pets and who are searching for pet foods that would mostly resemble their own food.

The objective of this review is to discuss the current data on traditional fiber sources utilized by the pet food industry and to explore the use of alternative dietary fiber sources in companion animal nutrition, as they may exert positive physiological effects when incorporated in companion animal foods.

## 2. Traditional Fiber Sources Used in Companion Animal Nutrition

Beet pulp and microcrystalline cellulose have been historically common fibers researched for use in and added to commercial pet foods in the US. They are included in pet foods, because they differ in their chemical composition and physiochemical properties, which determine fiber fermentability and affect physiological outcomes. A comparison of these traditional fibers can provide insight into the role of different fiber types in pet foods. Microcrystalline cellulose is a relatively non-fermentable, insoluble, non-viscous fiber [8,9,10]. There is little variation in the macronutrient composition of microcrystalline cellulose reported in the literature (Table 1). However, there are non-purified commercial sources of cellulose (e.g., wood cellulose) that include amorphous cellulose and other substances. These products may vary compositionally more than purified microcrystalline celluloses.

Beet pulp is a moderately fermentable fiber with viscous and non-viscous components [11,12]. It can be variable in macronutrient composition (Table 1), but often is high in pectin, cellulose and hemicelluloses. Fahey *et al.* [11] reported utilizing beet pulp containing 16% viscous polysaccharides (total dietary fiber (TDF) minus neutral detergent fiber), 31% hemicelluloses and non-viscous polysaccharides (neutral detergent fiber minus acid detergent fiber) and 25% cellulose (acid detergent fiber minus acid detergent lignin). Bosch *et al.* [13] reported similar values (22%) for the cellulose component of beet pulp; however, the concentration of hemicelluloses and non-viscous polysaccharides was lower (22%).

**Table 1:** Chemical composition of beet pulp and microcrystalline cellulose as reported in the literature.

Item	Beet Pulp	Microcrystalline Cellulose
Dry matter, %	87.6–92.3 [8,9,12,13,14,15,16]	93.0–96.6 [8,9,12,14,16,17]
Organic matter, %	90.4–95.4 [8,9,11,12,13,14,15,16]	99.4–100 [8,9,12,14,16,17]
Crude protein, %	7.5–16.3 [8,9,11,12,13,14,15,16]	0–2.0 [8,9,12,14,16,17]
Total dietary fiber, %	57.0–82.6 [8,9,11,12,16,18,19]	91.6–99.9 [8,9,12,14,16,17]
Insoluble dietary fiber, %	46.9–68.9 [14,16]	92.0–97.0 [14,16,17]
Soluble dietary fiber, %	13.1–28.6 [14,16]	2.3–3.5 [14,16,17]
Insoluble:Soluble dietary fiber	1.9–5.3:1 [14,16]	27.5–42.2:1 [14,16,17]

These data highlight the variation that can occur in plant by-products, which ultimately determine their physicochemical properties and fermentability. In general, cellulose, hemicelluloses and lignin are non-viscous, and pectins and gums are viscous. Pectic substances and gums are easily fermented, whereas fermentability of hemicelluloses and celluloses also depend on solubility and crystallinity [20,21]. The composition of plant products is impacted by soil and environmental conditions during growth, maturity at harvest, harvest date, plant parts included and preparation of plants. There is a need to chemically analyze fibers present in by-products rather than utilizing tabular values for diet formulations.

The fiber characteristics discussed above also impact *in vitro* fermentation and *in vivo* macronutrient digestibility [8,9,10]. Sunvold *et al.* [8,9,10] compared fermentability of cellulose and beet pulp in dogs and cats utilizing a 24-h *in vitro* organic matter disappearance (OMD) assay and *in vivo* TDF digestibility (diets contained 9 to 11% TDF). Fermentability of beet pulp, as assessed by *in vitro* OMD utilizing dog and cat inocula, was estimated to be 33 to 38% and 35 to 42%, respectively [8,9,10]. In comparison, cellulose had 2 to 4% and 0 to 1% *in vitro* OMD when dog and cat inocula were utilized, indicating low fermentability. These fermentation characteristics were reflected in the *in vivo* data; dogs fed diets containing beet pulp as the primary fiber source had higher (*p* < 0.05) apparent total tract TDF digestibility (29%) compared to those fed diets containing cellulose (11%) [9]. Cats fed diets containing beet pulp as the primary fiber source had higher (*p* < 0.05) apparent total tract TDF digestibility (38%) compared to those fed diets containing cellulose (9%) [8]. Fermented fibers provide substrates for short chain fatty acid production by microflora in the large bowel, while non-fermented fiber sources improve bowel health by promoting laxation, reducing transit time and increasing stool weight [8,9,10].

The influence of dietary fiber inclusion on the digestibility of other macronutrients and energy is more complex, and there are many factors that contribute to the influence of dietary fiber on macronutrient and energy digestibility coefficients. Firstly, ileal and fecal sample composition is, in part, a reflection of the nutrient composition, digestibility and fermentability of the fiber itself. For example, microcrystalline cellulose has high dry matter (DM) and organic matter (OM) concentrations and low digestibility and fermentability (*i.e.*, it travels through the gastrointestinal tract (GIT) relatively unchanged). Thus, depending on the dietary inclusion level, decreased apparent total tract DM and OM digestibilities are expected. In cats, compared with those fed a 0% supplemental fiber control (TDF: 1.7%), inclusion of 7.5% as-is cellulose (TDF: 11.2%) decreased (*p* < 0.05) digestibility of DM (88% *vs.* 81%) and OM (91% *vs.* 84%). Similar digestibility coefficients and different DM and OM digestibilities among treatments have been reported for dogs fed diets containing 2.5% cellulose (TDF: 5.0%) and 7.5% cellulose (TDF: 9.7%) when compared to those fed 0% supplemental fiber control diets (TDF: 2.5%) [22,23].

Dietary fibers also can impact digestibility or fermentability of other dietary ingredients. There are many reasons for this, including effects on nutrient intake, digesta transit time, sequestration of nutrients and more. Of particular interest is the impact of fibers on protein (N) digestibility. Much of the protein that escapes digestion in the small intestine is fermented in the large bowel [24]. While this fermentation produces beneficial short-chain fatty acid (SCFA), it can also produce putrefactive compounds (e.g., H_2_S, indolic and phenolic compounds, branched-chain fatty acid (BCFA)). Additionally, if significant amounts of protein escape digestion, its fermentation can alter the environment to favor the proliferation of potentially pathogenic species [25]. Muir *et al.* [22] examined apparent ileal and total tract macronutrient digestibility by dogs fed a 0% supplemental fiber control (TDF: 2.6%) compared to four fiber treatments (added at 7.5% as-is): beet pulp (TDF: 8.6%); 5% pectin + 2.5% cellulose (TDF: 9.7%); 2.5% pectin + 5% cellulose (TDF: 9.7%); and cellulose (TDF: 8.7%). They reported no impact of fiber treatments (*i.e.*, no fiber *vs.* four fiber treatments combined) on ileal digestibility. Middelbos *et al.* [23] reported similar data for 2.5% cellulose + 2.5% beet pulp diets compared to a 0% supplemental fiber control diet for dogs.

More data are available on total tract crude protein digestibility; however, results are conflicting, with some researchers reporting decrease in apparent total tract N digestibility for beet pulp diets compared to a 0% supplemental fiber control diet or cellulose diets [9,11,14,18] and others reporting no differences [8,15,19,23]. Results are potentially due to differences among studies in fiber inclusion levels, interactions between dietary matrix and the fiber and the numbers of animals utilized. The dietary inclusion of beet pulp or other fermentable fibers, and the subsequent fermentation in the colon, may enhance the fermentation of protein or enhance production of microbial N due to increased energy availability. By providing energy, fermentable fibers encourage microbial growth and, thus, contribute to the production of nitrogenous constituents. The impact of fermentable fibers on fecal microbial N in dogs and cats has not been extensively evaluated; however, decreases in urinary N linked to the inclusion of dietary fiber and increased fecal N excretion have been reported for both dogs and cats [26,27,28].

## 3. Alternative Dietary Fiber Sources in Companion Animal Nutrition

### 3.1. Corn Fiber

Corn fiber is the most abundant low value co-product from the corn wet milling industry [29]. Typically, a bushel of corn yields 2.04 kg of corn fiber. On average, the crude protein content of corn fiber is around 8.4%, total dietary fiber, 85.5%, and crude fat, 0.9% [30]. However, modifications of the wet milling process can impact the chemical composition of the corn fiber and may result in different physicochemical properties and physiological effects once incorporated into diets and ingested by pet animals.

Coarse fiber (from the pericarp) and fine fiber (mainly from the endosperm) are the two fiber types produced from a wet milling plant. In relation to fine fibers, coarse fiber has comparatively higher concentrations of total phenolic acids, lipids and arabinoxylans [29,31]. These coarse fiber components may have excellent emulsifying properties [29]. Further potential health benefits associated with the presence of phenolic compounds in corn fiber are their action as anti-mutagens, reducing the risk of colon cancer, and their antioxidant effect [32]. Corn fiber also may exert a prebiotic effect if the arabinoxylans are present in the form of oligosaccharides [31]; however, this remains to be investigated in companion animals.

Little research has been done using corn fiber as an ingredient in the diets of companion animals. However, because of its low cost, relatively high abundance (with increased ethanol production) and nutritional characteristics, it is important to investigate whether corn fiber can be successfully used in companion animal diets and whether its quality is comparable to the standard fiber sources used by the industry. De Godoy *et al.* [33] determined the chemical composition and *in vitro* fermentation characteristics of three corn fibers (two commercially available corn fiber products and a novel corn fiber produced without use of sulfur dioxide during the wet milling process). The corn fiber sources showed similar chemical composition. On a DM basis, corn fibers contained 71.4%–82.2% TDF, 5.0%–6.0% acid hydrolyzed fat (AHF), 7.5%–11.0% crude protein (CP) and 0.8%–0.9% ash. In contrast, beet pulp (positive control) had a higher ash concentration (6.8%) and lower TDF (68.8%) and CP (6.3%) concentrations, whereas cellulose (negative control) was comprised entirely of TDF (100%). The low ash content of corn fibers and the high concentration of TDF favor their utilization in pet food matrices, resulting in little interference with other nutrient categories, especially ash, where a maximum concentration needs to be guaranteed on the pet food label. Organic matter disappearance (OMD) after *in vitro* hydrolytic digestion of corn fibers varied from 6.5% to 22.0% (*p* < 0.05). Beet pulp had an OMD of 20.5%, whereas OMD values for cellulose and peanut hulls were 0.0% and 3.3%, respectively. The higher OMD values observed during hydrolytic digestion for one of the commercial corn fiber sources and for beet pulp were probably related to the presence of non-structural polysaccharides. After 16 h of *in vitro* fermentation using canine fecal inoculum, corn fibers were poorly fermented, with OMD ranging from 3.0% to 5.7%, in contrast to 17.7% for beet pulp and 0.0% for cellulose. The chemical composition and *in vitro* fermentation data suggest that corn fibers can be potentially used in pet foods, and they behave mostly as insoluble, non-fermentable fibers [33].

Another study examined the chemical composition, *in vitro* fermentation characteristics and *in vivo* nutrient digestibility of fiber-rich corn co-products: native corn fiber (wet milled corn pericarp), native corn fiber with fines (90% wet milled corn pericarp and 10% fine corn fiber particles), hydrolyzed corn fiber (native corn fiber subjected to steam injection followed by removal of solubilized hydrolysate) and hydrolyzed extracted corn fiber (hydrolyzed corn fiber extracted with ethanol) in adult dogs [31]. In general, a similar chemical composition to the corn fibers of the aforementioned study was observed herein. On a DM basis, CP ranged from 10.8% to 14.1%, TDF varied from 63.0% to 88.2% and AHF from 2.4% to 6.8%. The native corn fiber with fines had the lowest TDF and highest CP concentrations; this could be explained by higher concentrations of non-structural polysaccharides (e.g., starch) and remnants of CP from the endosperm present in the fines. *In vitro* OMD during the hydrolytic-enzymatic digestion step ranged from 7.2% to 31.1%, being greatest for native corn fiber with fines and lowest for hydrolyzed extracted corn fiber (*p* < 0.05). After 16 h of *in vitro* fermentation, native corn fibers showed intermediate fermentation (average, 9.6%), while fermentation of the hydrolysable corn fibers was negligible in contrast to beet pulp (17.7%; *p* < 0.05). When 7% of the corn fiber sources were added to dog food to replace beet pulp, no negative effects on food intake, nutrient digestibility or fecal quality were observed [31].

*In vitro* hydrolytic digestion, glycemic and insulinemic responses and true metabolizable energy using canine and avian models have been measured on a series of soluble corn fibers originating from different processing methods: hydrochloric acid and (or) phosphoric acid catalyzation, hydrogenation and spray-drying [34]. Among the soluble corn fibers, glucose was the primary free and bound monosaccharide after *in vitro* hydrolytic digestion, except for the hydrogenated fiber source, which had a greater concentration of sorbitol. In addition, processing method had a major impact on *in vitro* hydrolytic digestion of these substrates. In general, spray-dried, hydrogenated and phosphoric acid-treated soluble corn fibers were more digestible (~47%) than the fibers produced by hydrochloric acid or the combination of phosphoric and hydrochloric acids (29%). Soluble corn fibers, when orally provided to adult dogs, resulted in lower glycemic and insulinemic responses when compared with maltodextrin (*p* < 0.05), a highly digestible and rapidly absorbable carbohydrate used as a positive control. In agreement with the glycemic response, all soluble corn fibers had lower (1.3–3.0 kcal/g) true metabolizable energy in contrast to maltodextrin (4.1 kcal/g; *p* < 0.05) [34]. A similar study examined the effects of blends of soluble corn fibers with pullulan and sorbitol, both slowly digestible carbohydrate sources, and fructose, a non-insulinemic sugar [35]. In this study, soluble corn fiber had an *in vitro* hydrolytic digestion of approximately 50%. Blending soluble corn fiber with a low concentration (5% or 15%) of fructose resulted in similar monosaccharide digestibility values. However, blending soluble corn fiber with 30% or 50% fructose, sorbitol or pullulan led to greater digestibility, up to 91%. Soluble corn fiber and its blends had lower glycemic and insulinemic responses than maltodextrin. The lowest glycemic response was observed for blends containing 30%–50% fructose or sorbitol, resulting in an average relative glycemic response of 4.8% in contrast to maltodextrin (100%) [35]. Similar to soluble corn fiber, corn-based soluble fiber dextrin, produced by submitting corn starch to a thermal, chemical and enzymatic treatment, has been shown to lower glycemic and insulinemic responses by as much as 27% and 20%, respectively, in adult dogs and to have lower true metabolizable energy (37%) using the cecectomized rooster model when compared to maltodextrin [36]. Overall, corn fiber sources are good candidate ingredients to be incorporated into reduced glycemic and caloric canine diets.

In addition to the lower digestibility and glycemic response, soluble corn fiber also has been shown to positively modify indices of health in the cecum and colon of Sprague-Dawley rats [37]. Rats fed for 21 days a diet containing 5% soluble corn fiber, 5% soluble dextrin fiber or 5% pectin had increased crypt depth, goblet cell numbers and acidic mucin when compared to rats fed a control diet (5% cellulose, *p* < 0.05). Increased crypt depth is associated with increased rate of cell turnover and differentiation. In addition, goblet cells are responsible for mucus synthesis and secretion, which confers a protective barrier on the intestinal mucosa and prevents bacterial translocation, especially of sulfomucin-producing (acidic) [37]. Additionally, soluble corn fiber has been shown to decrease the concentration of putrefactive compounds (BCFA, indole and ammonia) and to increase the concentration of Bifidobacteria spp. in adult male subjects and *in vitro* [38,39].

### 3.2. Fruit Fibers

Fruit fibers and pomaces are by-products of the processing of fruits to juice or puree that are dried and, to some extent, further processed and ground to a fine particle size [40]. A general characteristic of fruit fibers is their higher content of pectin and hemicelluloses in relation to cellulose, accompanied by low fat and protein contents (<1%) [41]. Aside from the balanced profile of soluble to insoluble fiber, fruit-based products have good water-binding properties that can be used in food processing to control food texture and rheological behavior [41]. The latter attribute may be advantageous in wet pet food diet matrices, where high water content is required, but low water activity and firm texture is desirable. Additional benefits as regards utilization of fruit fibers in companion animal nutrition include the presence of bioactive components (e.g., flavonoids), a constant and inexpensive alternative fiber source and positive tag appeal on pet food labels. These factors have made fruit fibers an attractive ingredient in pet nutrition and increased their popularity among pet owners and pet food companies.

Chemical composition and fermentative characteristics of several fruit pomaces were studied using a canine *in vitro* model [17]. Total dietary fiber concentration varied among the different pomace sources; apple pomace had the greatest TDF concentration (79%), whereas grape pomace had the lowest (55%), tomato pomace and fruit blend (mixture of peach, almond, nectarine and plum) had intermediate values, 57% and 65%, respectively. Tomato and grape pomaces had a greater ratio of insoluble:soluble fiber (13:1 and 11:1, respectively) in contrast to apple pomace, which had the lowest ratio, 6:1. In general, fruit fibers with a greater insoluble:soluble fiber ratio had lower gas production and SCFA production after 12 or 24 h of *in vitro* fermentation. After 24 h of fermentation, apple pomace had a greater total SCFA concentration (2.1 mmol/g) in contrast to grape pomace, which had the lowest concentration (0.83 mmol/g) [17]. Sunvold *et al.* [12] evaluated *in vitro* fermentation characteristics of several dietary fiber sources, including citrus pulp and citrus pectin, using fecal inoculum from cats, dogs, horses, pigs, humans and cattle. Across species, citrus fibers had the greatest organic matter disappearance (OMD) (>80%) and total SCFA production (>5.5 mmol/g substrate OM). Surprisingly, when data pooled across all fiber substrates (cellulose, beet pulp, citrus pulp and citrus pectin) and fermentation times (6, 12, 24 and 48 h) were compared, the cat had the greatest total SCFA production, 3.38 mmol/g substrate OM, whereas the horse had the lowest, 1.61 mmol/g of substrate OM. These data disprove the concept that as a strict carnivore, cats are unable to utilize and benefit from dietary fibers [12,42]. Previous studies have reported similar OMD and SCFA production for citrus pectin using cat and dog fecal inoculum in *in vitro* models [8,9,10].

Another study investigated the effect of apple pomace inclusion in a meat-based diet for adult cats [43]. In this study, apple pomace was added at a ratio of 10, 20 or 40% of the diet. Increasing levels of apple pomace significantly decreased nutrient digestibility, especially DM (from 81% to 57%), OM (from 85% to 57%) and CP (from 86% to 69%), when compared to a control diet [43]. The decrease in fat digestibility was not as severe as observed for other nutrients, varying from 99% in the control diet to 94% in the 40% apple pomace diet. This is a relevant finding, since dietary fat is the primary source of energy for cats. Inclusion of apple pomace up to 20% of the diet did not decrease food palatability; however, at the 40% level, it resulted in lower food intake (*p* < 0.05). The data indicate that apple pomace is a palatable fiber source for adult cats up to a 20% inclusion level and that it can be used to reduce the caloric density of cat food. However, lower inclusion levels (e.g., 10% or 20%) would be most appropriate. In addition, the reduced nutrient digestibility, especially CP digestibility, should be taken into consideration when formulating diets with high levels of apple pomace.

Although the use of fruit and fruit fibers in companion animal nutrition is steadily receiving more attention from the pet owner and the pet food industry, studies related to health benefits associated with fruit fibers are sparse. Therefore, future research should investigate the potential positive effects of fruit fibers, not only related to their effects on nutrient digestibility and palatability, but also examining the latent beneficial effects of their phytochemicals that function as antioxidants, phytoestrogens and anti-inflammatory agents in promoting health or mitigating disease (e.g., obesity and its co-morbidities) of companion animals, as well as possible beneficial effects on hindgut microbiota.

### 3.3. Rice Bran

Rice (*Oryza sativa*) is an important cereal grain in global nutrition [44]. Approximately 631 million metric tons of rice are harvested annually worldwide [45]. Most of the rice produced and processed is used in human nutrition. When paddy rice undergoes the milling process, the hull is the outermost layer and the first to be removed, resulting in brown rice that is considered a whole grain [45]. Further milling of brown rice to white rice removes the rice bran [44]. Over 63 million tons of rice bran are produced each year, and the majority of it (~90%) is utilized in animal feeding [45]. On average, the nutritional composition of rice bran ranges from 21% to 27% TDF (mostly insoluble), 12%–16% crude protein and 18%–22% crude fat [45]. An important consideration as regards utilization of rice bran in animal and human nutrition is the stabilization of this ingredient by heat treatment that inactivates the lipase activity present in the rice seed coat, avoiding lipid oxidation and the formation of off flavors and odors [44].

Several bioactive molecules also are found in rice bran. Among them, phytochemicals, such as tocopherols, tocotrienols, polyphenols (ferulic acid and α-lipoic acid), phytoesterols, γ-oryzanol and carotenoids (carotene, lycopene, lutein and zeazanthin), have strong antioxidant, anti-inflammatory and chemopreventive properties and have potential efficacy in the management or prevention of chronic diseases [44]. In addition, rice bran oil contains a good fatty acid profile of mostly mono- and poly-unsaturated fatty acids—oleic acid (38.4%), linoleic acids (34.4%) and α-linolenic acid (2.2%) [46]—and about 1.5% γ-oryzanol, which has a strong antioxidant capacity. Rice bran also is a good source of essential amino acids, mainly sulfur amino acids, and micronutrients, including magnesium, manganese and B-vitamins [44].

In animal feeding, rice bran and fat may comprise up to 40% of dietary intake of pigs, cows and poultry [44]. In companion animal nutrition, brewer’s rice, brown rice and rice bran are ingredients commonly used in pet foods. However, the literature on the potential health benefits of rice bran is still scarce. Spears *et al.* [47] evaluated the diet palatability, nutrient digestibility, fecal characteristics, blood lipid profile and selected immune mediators in dogs fed dry pet foods containing 12% stabilized rice bran (produced by the inactivation of lipase) or defatted rice bran. In this study, inclusion of 12% stabilized or defatted rice bran was well tolerated by dogs, showing no detrimental effect in diet nutrient digestibility, fecal characteristics or changes in inflammatory immune mediators. In addition, the stabilized rice bran-containing diet had a greater palatability than the diet containing the defatted rice bran [47].

More recently, the fermentative profile of rice bran, alone and in combination with probiotics (*Lactobacillus acidophilus* 1415B or *Bifidobacterium longum* 05) was evaluated in an *in vitro* study using canine fecal inoculum in a stirred, pH-controlled and anaerobic batch culture system [48]. At 24 h of fermentation, rice bran resulted in greater concentrations of lactate, propionate and butyrate in comparison with rice bran in combination with one of the probiotics or probiotic treatment alone. In addition, fermentative vessels containing only rice bran had greater mean numbers of bifidobacteria and lactobacilli cells without a synergistic effect when rice bran was combined with probiotics. This study indicates that rice bran may be an economical alternative to prebiotic supplementation of pet foods [48].

To our knowledge, only one study has examined the effects of inclusion of rice bran in diets of adult cats. Stratton-Phelps *et al.* [49] reported that inclusion of 26% full-fat rice bran in a purified feline diet led to a significantly lower mean whole blood taurine concentration in comparison to a control group fed a purified diet containing 26% corn starch. At week 12 of the dietary treatment and thereafter (40 week), whole blood taurine concentration remained below the critical concentration (<200 nmol/L) for animals on the rice bran diet in contrast to the control group. The authors speculated that the lower taurine concentration observed in cats fed the rice bran diet was due to an increased fecal excretion of conjugated bile acids in addition to changes in hindgut microbiota due to the indigestible protein fraction of rice bran and that were able to degrade taurine (since this amino acid is not degraded by mammalian tissues). Based on this outcome, a higher concentration of dietary taurine (>0.05%) should be included in feline diets that contain rice bran. However, a quantitative relationship between the dietary inclusion level of rice bran and the taurine adequacy in feline diets still needs to be determined [49].

A recent *in vitro* study examining the anti-cancer activity of rice bran phytochemicals in colorectal cancer cells demonstrated that total phenolics and γ-tocotrienol were positively correlated to reduced cancer cell growth [50]. However, a diverse profile and concentration of phytochemicals were observed among different rice bran varieties [50]. Future studies evaluating the potential health benefits of rice bran should emphasize nutrient-host-microbiome interactions and changes in metabolic profile of disease markers of chronic illness that rice bran has potential to be effective against [44].

### 3.4. Whole Grains

According to the American Association of Cereal Chemists International and the FDA, whole grains are defined as the “intact, ground, cracked or flaked fruit of the grain whose principal components, the starchy endosperm, germ and bran, are present in the same relative proportions as they exist in the intact grain [51,52]”. Whole grains are comprised mostly of endosperm (~80%), with the germ and bran making up variable proportions among different grains [53]. In human nutrition, wheat, corn, oats, barley and rye are the most popular sources of whole grains [53]. Nutritionally, whole grains are rich in dietary fiber, trace minerals and vitamins B and E [54]. In addition, whole grains are rich in bioactive compounds, such as phytochemicals (e.g., lignans, tocotrienols and polyphenols), antinutrients (e.g., phytic acid, tannins and saponins), lipotropes and methyl donors (e.g., betaine, choline, methionine, inositol and folate). The antioxidant, anti-carcinogenic and lipotropic effects of these compounds have shown a protective effect against chronic diseases, such as obesity, diabetes, cardiovascular and some forms of cancers [53,54,55,56].

In companion animal nutrition, corn, wheat and their co-products are ingredients commonly used in pet food formulations. However, more recently, their incorporation into pet foods has been perceived as negative by some pet owners who believe that a grain-free diet is a more adequate nutritional strategy for dogs and cats, due to their carnivorous nature, even though, to date, no scientific evidence supports this anecdotal belief. Paradoxically, while many pet food labels advertise “no corn” and “no wheat”, pet owners have shown increased interest in diets that are holistic, natural and that contain wholesome ingredients, of which oats and barley have become very appealing and well-accepted by pet owners.

Oats (*Avena sativa*) and barley (*Hordeum vulgare*) are two cereal grains that are good sources of β-glucan, a water-soluble fiber fraction that has plasma lipid- and glycemic-lowering effects in humans and animal models. Similar to humans, companion animals also have a high incidence of chronic diseases. Therefore, the use of oats and barley as functional ingredients in pet foods may be beneficial in the control or prevention of obesity, diabetes mellitus and dyslipidemia.

### Role of β-Glucans in Companion Animal Nutrition

Cereal β-glucans are classified as soluble dietary fibers and have rheological properties comparable to guar gum and other random coil polysaccharides [57]. Typically, the β-glucan content of oats and barley ranges between 3%–7% and 5%–11%, respectively [58], and is an important component of the cell wall of these cereal grains [59]. The molecular arrangement of β-glucans consists of d-glucose molecules connected by a series of β-(1→3) and β-(1→4) linkages [60,61]. The viscous behavior of these non-starch polysaccharides is related to their physical arrangement; the presence of β (1→3) linkages leads to bends in the straight chain of the polymer allowing water to permeate and conferring high water solubility and viscosity properties [62,63].

The ability of β-glucans to form highly viscous solutions has been associated with their beneficial physiological effects [56]. Wood *et al.* [59] demonstrated that the viscosity of β-glucans accounts for 79%–96% of the changes in glycemic and insulinemic responses. In addition, the molecular weight (MW) and concentration, the nature of the extract, the form of delivery and the dose ingested can also influence the bioactivity of β-glucans [57,64,65]. Biorklund *et al.* [66] reported that oat β-glucan with a MW of 70,000 lowered serum cholesterol and postprandial glucose and insulin concentrations, whereas barley β-glucan with a lower MW of 40,000 did not. In addition, thermal treatment—cooking and freezing processes—may modify the rheological properties of β-glucan, decreasing its solubility [67,68]. This is a factor that should not be disregarded by pet food manufacturers, because of the harsh thermal treatments (e.g., extrusion and canning) applied to their products that may decrease the bioactivity of β-glucans. In addition, other factors may play a role in the animal’s physiological response to β-glucan intake (age, gender and physiological status [69]).

Literature available on the effects of cereal β-glucans on nutrient digestibility by dogs is limited. A study reported that inclusion of 40% extruded barley to a basal diet, predominantly made of corn, wheat and animal fat, resulted in decreased fecal dry matter and, consequently, looser stools. Calculated barley nutrient digestibility was high for organic matter (92%) and for nitrogen free extract (98%), whereas CP digestibility was lower (71%) [70]. Lower apparent CP digestibility by dogs fed diets containing soluble dietary fibers has been attributed to an increased microbial mass, due to increased fermentative activity in the large intestine [71].

Other mechanisms have been proposed in order to explain how β-glucans may positively or negatively impact nutrient digestibility and absorption. Improvements in nutrient digestibility could result from delayed gastric emptying rate and longer mean retention times induced by increased viscosity of the digesta. This would extend the contact of the nutrients in the gastrointestinal tract with digestive enzymes [72,73,74] or could be a reflection of a greater digestibility of dietary fiber, due to fermentation of β-glucans [75]. Conversely, decreased nutrient digestibility and absorption could indicate reduced digestive enzyme activity by non-specific binding of the enzymes or the presence of specific enzyme inhibitors [76].

Consumption of β-glucan improved glycemic and (or) insulinemic response in healthy humans [3,77,78,79] and in obese and type II diabetic patients [80,81]. In contrast, other studies failed to identify improvements in insulin response by the dietary supplementation of β-glucan using human [82] and animal models [83,84]. The effect of β-glucan in blunting glycemic response is possibly mediated by the delay in gastric emptying, resulting in slower and gradual absorption of glucose. In adult dogs, incremental dietary levels of barley (10, 20 or 40% at the expense of yellow corn) have been evaluated for apparent nutrient digestibility, fecal fermentation end-products and postprandial glycemic and cholesterolemic responses [85]. Increasing levels of dietary barley had no effect on apparent DM digestibility (average 87%) or fecal score (average 2.9 on a five-point scale with three being considered ideal); however, a quadratic effect was observed for apparent TDF and AHF digestibilities. A mean apparent TDF digestibility of 68% was observed for animals fed the 40% barley diet in contrast to 50% for the control, with intermediate values observed for the 10 and 20% barley treatments (average, 55%). The opposite was observed for apparent AHF digestibility, with the control diet having a mean of 96% in contrast to 94% for both the 20%- and 40% barley treatments. A quadratic increase (*p* < 0.05) in total SCFA production also was observed with increasing levels of barley supplementation. However, no treatment effect was observed for postprandial plasma glucose or cholesterol concentrations (*p* > 0.05). These results indicate that inclusion of up 40% of barley in diets of adult dogs is well tolerated and does not cause detrimental effects on fat digestibility, as it was maintained within an acceptable range (>90%). Further studies should explore the effect of barley in obese, diabetic or hypercholesterolemic dogs and cats, as they might be a more responsive model to the potential health benefits associated with the consumption of β-glucans present in barley [85].

A recent study in adult dogs investigated the effects of consumption of complex carbohydrates (barley, corn, peas and rice) supplemented at a daily dose of 10 g of available carbohydrate on glycemic response and cardiovascular health and oxidative stress markers [86]. Among the carbohydrate sources, peas had the lowest glycemic index value (29%) compared to barley and rice (51 and 55%, respectively, *p* < 0.05). However, no differences in postprandial glucose peak (5.3 mmol/L) or time to peak (95.3 min) were observed among the complex carbohydrate sources, which were lower (*p* < 0.05) than the postprandial glucose peak (8.5 mmol/L) and time to peak (34 min) values observed for a glucose solution used as a positive control. Methylglyoxal, a marker of oxidative stress associated with consumption of a high glycemic index diet, was increased in dogs fed the glucose solution (140%) when compared with animals fed the complex carbohydrate sources (~95%). A decrease (*p* < 0.05) in flow-mediated dilation, an indicator of endothelial dysfunction, also was observed for animals fed the glucose solution (20 nM) in contrast to dogs on the complex carbohydrate treatments (~30 nM). Overall, this study suggests that consumption of complex carbohydrates may have a protective effect on cardiovascular health and oxidative stress related to hyperglycemia [86].

## 4. Concluding Remarks and Future Considerations

The use of alternative fiber sources in pet foods seems a promising and important area in companion animal nutrition, as growing evidence supports their beneficial effects in improving the health status of pets. In addition, corn and fruit fibers, rice bran and fibers rich in β-glucans are well tolerated by adult dogs and cats. The data gathered herein offer valuable information for the pet food industry, as well as pet owners seeking alternative fiber sources and nutraceutical ingredients as a mean of enhancing health and longevity of companion animals or to aid in the management of common maladies noted in today’s pet population: hyperlipidemia, insulin resistance, diabetes mellitus, *etc.* Further research is necessary to determine optimal levels of supplementation of these fiber sources in diets targeting select physiological states of dogs and cats, as well as in different diet matrices. A better understanding of the variation in chemical composition among different fiber sources, as well as their response to processing conditions, also will aid in revealing the bioactive components present in these ingredients that provide their nutraceutical properties. Future evaluation of nutrient-host-microbiome interactions also is warranted in order to advance our understanding of the physiological mechanisms by which phytochemicals present in fiber sources enhance health.

## List of Abbreviations

AHF: acid hydrolyzed fat
BCFA: branched-chain fatty acid
CP: crude protein
DM: dry matter
DRI: Dietary Reference Intakes
FDA: Food and Drug Administration
GIT: gastrointestinal tract
MW: molecular weight
N: nitrogen
OM: organic matter
OMD: organic matter disappearance
SCFA: short-chain fatty acid
TDF: total dietary fiber
